# Agreement Between Serum Assays Performed in ED Point-of-Care and Hospital Central Laboratories

**DOI:** 10.5811/westjem.2017.1.30532

**Published:** 2017-03-13

**Authors:** Meir Dashevsky, Steven L. Bernstein, Carol L. Barsky, Richard A. Taylor

**Affiliations:** Yale School of Medicine, Yale-New Haven Hospital, Department of Emergency Medicine, New Haven, Connecticut

## Abstract

**Introduction:**

Point-of-care (POC) testing allows for more time-sensitive diagnosis and treatment in the emergency department (ED) than sending blood samples to the hospital central laboratory (CL). However, many ED patients have blood sent to both, either out of clinical custom, or because clinicians do not trust the POC values. The objective of this study was to examine the level of agreement between POC and CL values in a large cohort of ED patients.

**Methods:**

In an urban, Level I ED that sees approximately 120,000 patients/year, all patients seen between March 1, 2013, and October 1, 2014, who had blood sent to POC and CL labs had levels of agreement measured between serum sodium, potassium, blood urea nitrogen (BUN), creatinine, and hematocrit. We extracted data from the hospital’s clinical information system, and analyzed agreement with the use of Bland-Altman plots, defining both 95% confidence intervals (CIs) and more conservative CIs based on clinical judgment.

**Results:**

Out of 163,661 patients seen during the study period, 14,567 had blood samples sent both for POC and CL analysis. Using clinical criteria, the levels of agreement for sodium were 98.6% (within 5mg/dL), for potassium 90.7% (0.5 mmol/L), for BUN 89.0% (within 5 mg/dL), for creatinine 94.5% (within 0.3 mg/dL), for hematocrit 96.5% (within 5 g/dL).

**Conclusion:**

Agreement between POC and CL values is excellent. Restricting the analysis to clinically important levels of agreement continues to show a high level of agreement. The data suggest that sending a serum sample to the hospital CL for duplicate assays is unnecessary. This may result in substantial savings and shorter ED lengths of stay.

## INTRODUCTION

Point-of-care (POC) testing has been used in various medical settings for rapid determination of a variety of laboratory values without confirmatory testing.^1^–^4^ However, duplication of the POC test with central lab (CL) testing often occurs.^5^ Many reasons have been suggested for duplicate testing, such as distrust of the POC result, preference for a “real test” and fear that consultants or admitting physicians would not rely on the POC values.^6^ In the ED, duplication of POC testing is inefficient and wastes time, resources, and manpower.^7^

Potential causes of disagreement between tests may be secondary to sample collecting/handling (pre-analytical) or be related to the machine/test itself (post-analytical). Post-analytical problems are rare, as most institutions have strict guidelines and testing prior to introducing a new POC test as well as regular monitoring of all laboratory testing, including POC, to ensure ongoing accuracy. There are also strict guidelines and regulations as outlined by CLIA (Clinical Laboratory Improvement Amendments of 1988)^8^ and the Joint Commission^9^ to ensure the quality of laboratory testing. Pre-analytical problems are more likely culprits of actual or perceived disagreement and generally are caused by not strictly following guidelines for obtaining blood specimens, rather than the POC device itself.^10^ Hemolysis may also occur in POC testing and may accurately reflect the content of the blood tube but not the physiologic state of the patient (i.e. hyperkalemia in a hemolyzed sample); this can occur in CLs as well, though it may be more easily identified.^6^

While multiple studies have examined the impact of POC testing in the emergency department (ED),^11^–^14^ there is limited data on the level of agreement between POC and CL testing in the ED concerning electrolytes and hematocrit values.^15^ Prior literature examining testing in the intensive care unit reports varying levels of agreement with overall small sample sizes.^16^ Critical to the adoption of POC testing and reduction of duplication through CL ordering is high quality data supporting good agreement between the two tests. This study was designed to measure the level of agreement between POC and CL results. Our hypothesis was that they would be sufficiently concordant to allow reduction of duplicate testing.

## METHODS

In an urban, Level I ED that treats approximately 120,000 patient visits/year, we examined the level of agreement between POC and CL values drawn simultaneously. The POC machine used was the iStat 1 wireless analyzer, MN: 300W by Abbott Point of Care Inc. The CL used the chemistry DDP analyzer by Roche, and for the complete blood count the CELL-DYN Sapphire by Abbott Diagnostics. For the purposes of this study, blood samples time-stamped within one hour by the CL and POC lab were considered to be simultaneous. All patients age 21 years or older treated from March 2013 to September 2014 who had blood sent to POC and CL labs had levels of agreement measured for serum sodium, potassium, blood urea nitrogen (BUN), creatinine, and hematocrit.

For each lab test, we defined, *a priori*, a range of agreement that we considered clinically sensible. The ranges of agreement were as follows: sodium 5 mEq/L, potassium 0.5 mEq/L, BUN 5mg/dL, creatinine 0.3mg/dL and hematocrit 5%. Clinical agreement was determined by a group of senior clinicians in our department. The group attempted to choose range values that would, if true, potentially impact clinical disposition, ordering diagnostic tests (creatinine), or place patients into a higher severity of electrolyte imbalance. For instance, 5mEq/L was chosen for sodium values, as a difference of this magnitude on either end of the normal sodium range 135–145mEq/L would place a patient in the moderate hyponatremia or hypernatremia category.^17^,^18^ A similar approach was taken for potassium,^19^ BUN,^20^ creatinine,^21^ and hematocrit.^22^

Data were extracted from the hospital’s clinical information system and exported to STATA (Version 13). We analyzed agreement with the use of Bland-Altman plots, defining both 95% confidence intervals (CI) and more conservative CIs based on clinical judgment.^23^–^25^

**Population Health Research Capsule**

What do we already know about this issue?

Point-of-care and central laboratory testing are often duplicated due to concerns of disagreement between the two testing modalities.

What was the research question?

Is there clinically significant disagreement between point-of-care and central laboratory testing?

What was the major finding of the study?

Agreement between point-of-care and central laboratory testing is excellent.

How does this improve population health?

High level of agreement between testing modalities can lead to reduced duplicate testing, eliminating waste.

The institution’s Human Investigations Committee approved this retrospective study, and informed consent from patients was waived.

## RESULTS

There were 163,661 patient visits in the study period, of whom 18,268 (11.2%) had at least one assay measured in both CL and POC labs. The mean age (±SD) for these 18,268 patients was 59.9±19.4 years; 50.3% were male. Further demographic details and visit characteristics are given in Table 1.

Out of 163,661 patient visits, 14,567 (8.9%) had blood samples sent both for POC and chemistry CL analysis; and 16,908 (10.3%) had POC hematocrit and CL hematocrit.

**Figure 1:**
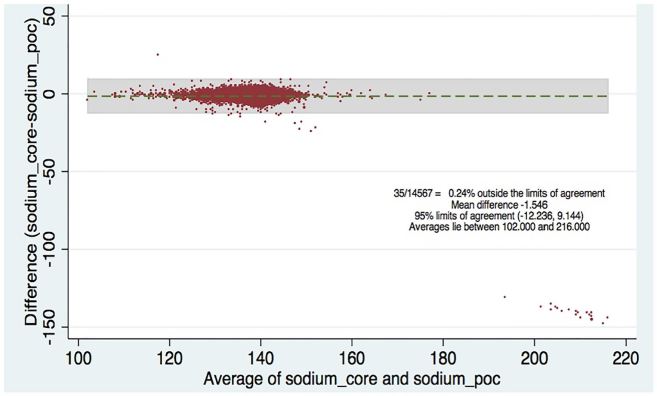
Bland-Altman plot for sodium. Comparing hospital central lab and emergency department point-of-care (POC) values.

**Figure 2:**
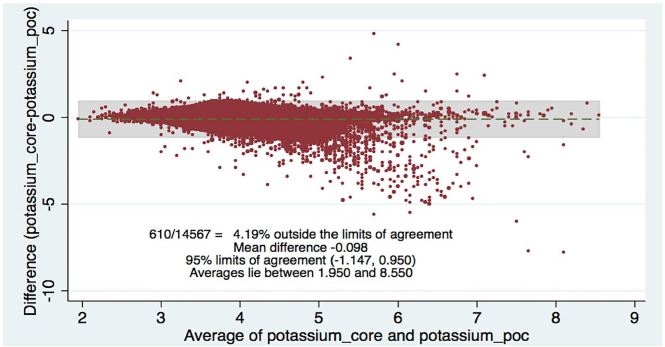
Bland-Altman plot of potassium. Comparing central lab vs. point-of-care values (POC) lab values.

**Figure 3:**
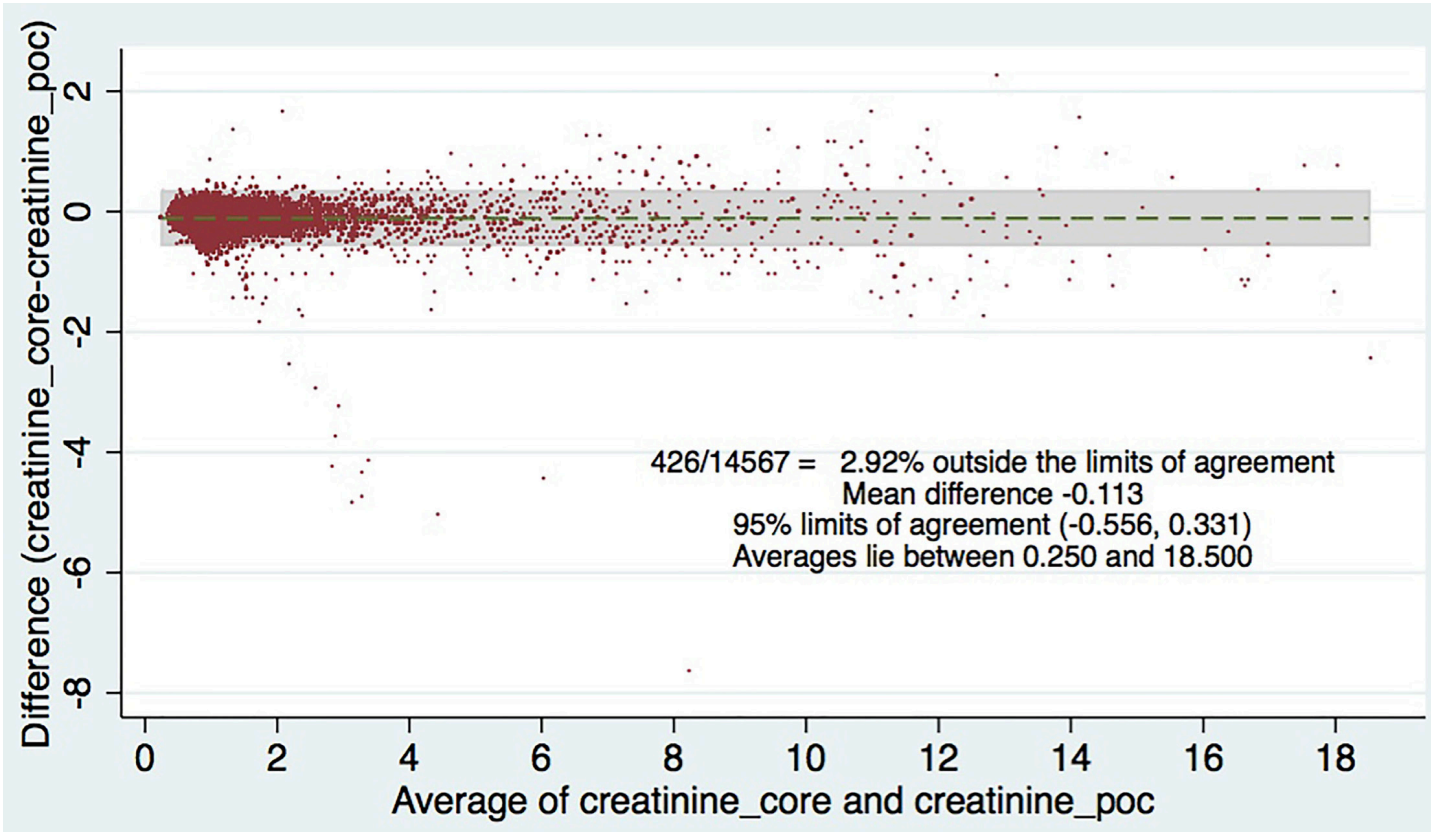
Bland-Altman plot of creatinine. Comparing central lab vs. point-of-care (POC) lab values.

**Figure 4:**
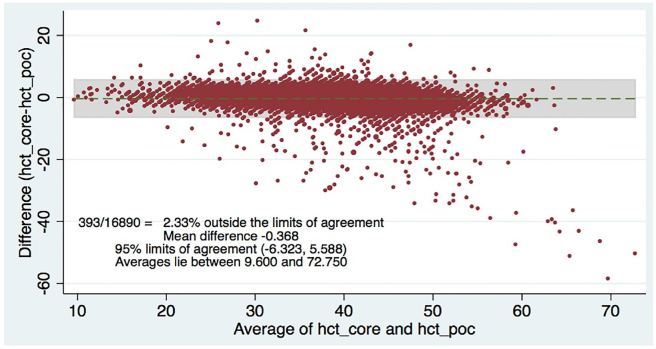
Bland-Altman plot for hematocrit (HCT). Comparing central lab vs. point-of-care (POC) lab values.

**Figure 5:**
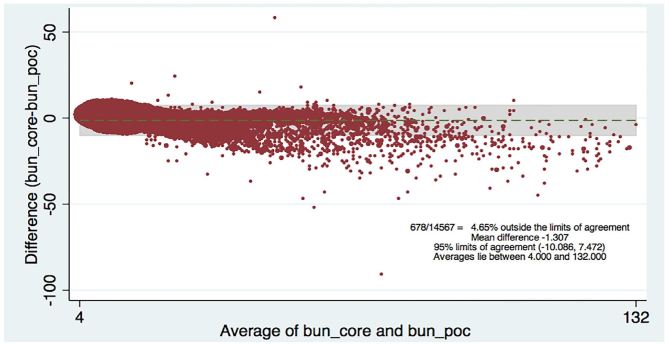
Bland-Altman plot of blood urea nitrogen (BUN). Comparing central lab vs. point-of-care (POC) lab values.

Table 2 shows the levels of clinical agreement: for sodium, 98.6%; potassium, 90.8%; BUN, 89.1%; creatinine, 94.6%; hematocrit, 96.5%. Figures 1–5 display the Bland-Altman plots for each test. The mean difference with 95% limits of agreement for each value is as follows: sodium −1.55 (−12.2, 9.1), potassium −0.10 (−1.1, 1.0), BUN −1.31 (−10.1, 7.5), creatinine −0.11 (−0.6, 0.3), hematocrit −0.37 (−6.3, 5.6).(Figures 1–5; Table 2).

## DISCUSSION

In a cohort of subjects treated over 19 months at a single hospital, we found very good agreement between POC and CL values. Though overall agreement was excellent, there is some variability between individual tests. Disagreement is more common at the extremes of lab ranges. Ranges of agreement were designed to highlight agreement within normal or near-normal values. In contrast, for grossly abnormal values, greater discordance is often not clinically relevant. For example, a difference of 1 mg/dL of creatinine from 1.0 to 2.0 is clinically significant, whereas the difference between creatinines of 7.0 and 8.0 is clinically unimportant. In this study, both sets would have been flagged as clinically important disagreement.

Sodium values had the highest degree of agreement. Interestingly, a subset (n=19) of the discordant labs resulted with POC sodium values ranging from >190–220. These results, clearly in error, were included in the analysis. It is unclear what caused these abnormal results; however, it is reasonable to assume that these values were immediately recognized as lab error and samples were either rerun or sent to the lab.

Potassium had a 90.25% percent of agreement. Most discordant pairs occurred when the POC result was in the high-normal to elevated range. Among these pairs, instances of lower POC potassium were exceedingly rare.

The percent agreement of BUN was the lowest at 89%. The clinical significance of this discordance is not clear. It is noteworthy that BUN was the only test in which the majority of the normal range disagreement resulted in lower POC than CL values.

The percentage agreement of creatinine was 94.6%. A window of 0.3 was chosen with the express purpose of detecting changes in the renal function that would be actionable.

Hematocrit values had an agreement of 96.53%. Insofar as hematocrit values are often used to determine indications for transfusions of blood products, the high degree of concordance is reassuring. Most discordant pairs occurred at values greater than 30, and thus are unlikely to affect decisions to transfuse.

The data indicate that values obtained from the hospital core lab should not be considered the criterion standard. POC values are sufficiently concordant for the blood tests we examined. Insofar as all blood tests are approximations of an *in vivo* phenomenon, some minimal level of variation is to be expected.

It is also important to note that different clinical scenarios might require ranges of agreement different from those used in this study. For example, a level of agreement of 98.64% at 5 mEq/L for sodium is sufficient for most clinical scenarios. However, in cases of severe hypo- or hypernatremia, the need for slow, precise correction over hours to days may require a tighter range for agreement. However, values from either the CL or POC appear sufficiently accurate and precise to manage scenarios of deranged sodium metabolism.

Our data suggest that, for the tests we studied, CL and POC testing are sufficiently concordant so that duplicate testing is unnecessary. If patient care or disposition is dependent on a timely value, then POC testing is preferred. If blood is being drawn as part of “routine” care for an admitted patient, then the CL might be preferred, insofar as the POC typically requires time and effort from ED nurses and nursing assistants. If any test result appears unexpectedly and critically abnormal, then it should be repeated, irrespective of which lab runs it.

Reduction of duplicate testing may result in significant savings of cost and effort, and improved patient flow through the ED. Based on our data (18, 268 duplicated studies in a 19-month time frame) and assuming a lab test cost of $14.37 for a chemistry study based on 2014 Medicare reimbursement values,^26^ the annual cost savings would be $165,796/year. By improving the efficiency and timeliness of care, reduction of duplicate testing enhances two of the domains of quality of care, defined by the Institute of Medicine.^27^

## LIMITATIONS

There are several limitations in this study. The retrospective design required the use of time-stamp proxies within the electronic medical record to locate the duplicate pairs. We chose one hour as a clinically sensible time range within which lab tests might be considered to yield comparable results. Another limitation is the use of time-stamp data as a proxy for time of venipuncture. For CL samples, the time represents the time of arrival in the lab; for POC samples, it represents the time the test was run. The difference between those two times is less than one hour, and likely within about 15 minutes. Hence, time stamp is likely a reasonable proxy for time of venipuncture.

Another limitation is the lack of universally agreed-upon ranges of clinically significant agreement. In this study, ranges of clinical agreement were determined by a group of experienced emergency physicians, with the goal of defining ranges that would not change practice. Others may define slightly different ranges of clinical concordance. Additionally, one might contend that although the proportion of agreement was high, it was still not sufficient. The Bland-Altman plots can provide additional insight into the clinical implications of discordance, particularly at very high absolute values.

This single-institution study may have limited generalizability. However, our sample is diverse with respect to age, gender, race, ethnicity, and insurance. Therefore, it is likely that these results would be similar elsewhere. In any event, we offer a method by which other institutions may assess the concordance between their POC and CL blood values.

Lastly, we did not perform an economic analysis to estimate the potential cost savings from decreasing or eliminating duplicate testing.

## CONCLUSION

We found a high level of clinical agreement for point-of-care and central lab chemistry tests. Duplicate ordering from POC and CLs may be unnecessary and wasteful. Using the data from this study, our institution has formed a clinical design team whose purpose is to eliminate unnecessary POC-CL testing. Education of clinicians, nurses and techs regarding study results and indications for testing is ongoing and the ordering workflow has been adjusted to further support these efforts. Further study of these efforts and the success of individual interventions is ongoing.

## Figures and Tables

**Table 1:** Characteristics of the sample of patients in a study comparing agreement between serum assays in the emergency department point-of-care setting vs. the hospital central lab.

Characteristic	N (%) 18,268
Age (yr) (mean, SD)	59.9 ± 19.4
Gender, n, (% male)	9179 (50.3)
Race	
White	11,043 (60.5)
Black	4,254 (23.3)
Other	2807 (15.4)
Asian	164 (0.90)
Insurance, n (%)	
Private/HMO	3,827 (20.9)
Medicaid	3,974 (21.8)
Medicare	8,471 (46.4)
Self-pay/uninsured	1,932 (10.6)
Other	64 (0.4)
Arrival by ambulance, n (%)	11,227 (61.5)
Triage acuity, ESI	
Level 1	1,098 (6.0)
Level 2	11,817 (64.7)
Level 3	5,096 (27.9)
Level 4	113 (0.6)
Level 5	4 (0.02)
ED disposition, n (%)	
Admit	13,003 (71.2)
Discharge	4935 (27.0)
Other	328 (1.8)

*HMO,* health maintenance organization; *ESI,* emergency severity index; *ED,* emergency department.

**Table 2:** Clinical agreement between central laboratory and ED point-of-care values for common blood tests

	Range	Agreement (%)
Sodium	5 mEq/L	98.64%
Potassium	0.5 mEq/L	90.75%
BUN	5 mg/dL	89.06%
Creatinine	0.3 mg/dL	94.55%
Hematocrit	5%	96.53%

*BUN,* blood urea nitrogen.
